# Supplementary material for: Feasibility of 4D-Spatio Temporal Image Correlation (STIC) in the Comprehensive Assessment of the Fetal Heart Using FetalHQ®
Source: J Clin Med. 2022 Mar 4;11(5):1414. doi: 10.3390/jcm11051414 (PMC8911117; doi:10.3390/jcm11051414)
Supplement: Supplementary file 1 [file jcm-11-01414-s001.zip › jcm-1576176-SI.pdf]

Journal of
Clinical Medicine

MDPI

Table S1. Intraobserver reproducibility of the fetal heart 4D-Spatio Temporal Image Correlation (4D-STIC) speckle tracking analysis results using Quiver technique.

Variable	ICC	95% confidence interval	*p*-value	ICC	95% confidence interval	*p*-value
**FETAL CARDIAC MORPHOMETRY**						
		Left Ventricle			Right Ventricle	
Ventricular Area	0.854	0.406 to 0.964	0.005	0.878	0.533 to 0.969	0.002
Longitudinal diameter	0.906	0.634 to 0.977	0.001	0.926	0.718 to 0.981	<0.001
Basal diameter (segment 1)	0.798	0.190 to 0.950	0.005	0.910	0.447 to 0.980	<0.001
Mid-ventricular diameter (segment 9)	0.681	−0.436 to 0.923	0.063	0.887	0.571 to 0.972	0.001
Apical diameter (segment 17)	0.549	−1.136 to 0.902	0.157	0.851	0.375 to 0.963	0.006
Basal sphericity index (segment 1)	0.244	−0.524 to 0.750	0.258	0.661	−0.128 to 0.911	0.034
Mid-ventricular sphericity index (segment 9)	0.616	−0.725 to 0.907	0.098	0.738	0.072 to 0.933	0.018
Apical sphericity index (segment 17)	0.432	−1.778 to 0.865	0.224	0.691	−0.387 to 0.925	0.058
**FETAL CARDIAC FUNCTION**						
		Left Ventricle			Right Ventricle	
Global longitudinal strain	0.983	0.936 to 0.996	<0.001	0.928	0.730 to 0.982	<0.001
Fractional area change	0.940	0.683 to 0.986	<0.001	0.883	0.562 to 0.970	0.002
Basal shortening fraction (Segment 1)	0.564	−0.239 to 0.886	0.018	0.726	−0.118 to 0.933	0.008
Mid-ventricular shortening fraction (Segment 9)	0.830	0.297 to 0.958	0.009	0.877	0.537 to 0.969	0.002
Apical shortening fraction (Segment 17)	0.687	−0.071 to 0.919	0.035	0.731	−0.191 to 0.935	0.040
End-diastolic volume	0.795	0.140 to 0.950	0.017			
End-systolic volume	0.545	−1.159 to 0.891	0.145			
Ejection fraction	0.920	0.5692to 0.980	<0.001			
Cardiac Output	0.898	0.585 to 0.977	0.002			

ICC: Intraclass Correlation Coefficient.

Table S2. Interobserver reproducibility of the fetal heart 4D-STIC speckle tracking analysis results using Quiver technique.

Variable	ICC	95% confidence interval	*p-value*	ICC	95% confidence interval	*p-value*
**FETAL CARDIAC MORPHOMETRY**						
		Left Ventricle			Right Ventricle	
Ventricular Area	0.989	0.790 to 0.998	<0.001	0.946	0.789 to 0.987	<0.001
Longitudinal diameter	0.952	0.806 to 0.988	<0.001	0.938	0.636 to 0.986	<0.001
Basal diameter (segment 1)	0.786	0.204 to 0.946	0.015	0.986	0.948 to 0.997	<0.001
Mid-ventricular diameter (segment 9)	0.959	0.836 to 0.990	<0.001	0.837	0.312 to 0.960	0.008
Apical diameter (segment 17)	0.825	0.267 to 0.957	0.010	0.886	0.566 to 0.971	0.001
Basal sphericity index (segment 1)	0.011	−1.562 to 0.715	0.492	0.696	−0.202 to 0.924	0.048
Mid-ventricular sphericity index (segment 9)	0.507	−1.371 to 0.882	0.171	0.578	−0.912 to 0.898	0.121
Apical sphericity index (segment 17)	0.260	−2.088 to 0.818	0.334	0.598	−0.806 to 0.903	0.110
**FETAL CARDIAC FUNCTION**						
		Left Ventricle			Right Ventricle	
Global longitudinal strain	0.907	0.621 to 0.977	0.001	0.713	−0.239 to 0.935	0.051
Fractional area change	0.796	0.133 to 0.950	0.017	0.938	0.753 to 0.985	<0.001
Basal shortening fraction (Segment 1)	0.119	−0.275 to 0.608	0.309	0.491	−1.451 to 0.878	0.182
Mid-ventricular shortening fraction (Segment 9)	0.308	−2.401 to 0.836	0.309	0.629	−0.690 to 0.910	0.091
Apical shortening fraction (Segment 17)	0.759	0.140 to 0.938	0.016	0.359	−1.863 to 0.845	0.269
End-diastolic volume	0.986	0.930 to 0.997	<0.001			
End-systolic volumen	0.948	0.787 to 0.987	<0.001			
Ejection fraction	0.668	−0.501 to 0.920	0.070			
Cardiac Output	0.925	0.679 to 0.983	<0.001			

ICC: Intraclass Correlation Coefficient.

Table S3. Comparison of 2D vs 4D-STIC Interobserver reproducibility of the fetal heart speckle tracking analysis results using Quiver technique.

Variable	ICC	95% confidence interval	*p-value*	ICC	95% confidence interval	*p-value*
**FETAL CARDIAC MORPHOMETRY**						
		Left Ventricle			Right Ventricle	
Ventricular Area	0.930	0.728 to 0.982	<0.001	0.964	0.864 to 0.991	<0.001
Longitudinal diameter	0.946	0.779 to 0.987	<0.001	0.965	0.806 to 0.955	<0.001
Basal diameter (segment 1)	0.802	0.264 to 0.950	0.011	0.949	0.805 to 0.987	<0.001
Mid-ventricular diameter (segment 9)	0.888	0.554 to 0.972	0.002	0.930	0.735 to 0.982	<0.001
Apical diameter (segment 17)	0.966	0.863 to 0.992	<0.001	0.935	0.745 to 0.984	<0.001
Basal sphericity index (segment 1)	0.347	−1.872 to 0.841	0.276	0.607	−0.553 to 0.902	0.093
Mid-ventricular sphericity index (segment 9)	0.662	−0.283 to 0.915	0.061	0.487	−1.117 to 0.874	0.176
Apical sphericity index (segment 17)	0.434	−1.730 to 0.865	0.221	0.236	−3.642 to 0.838	0.368
**FETAL CARDIAC FUNCTION**						
		Left Ventricle			Right Ventricle	
LV Global longitudinal strain	0.763	0.149 to 0.939	0.017	0.959	0.837 to 0.990	<0.001
LV Fractional area change	0.791	0.241 to 0.947	0.010	0.889	0.540 to 0.972	0.002
FS Segment 1 (base)	0.096	−2.755 to 0.793	0.444	0.806	0.227 to 0.951	0.011
FS Segment 9 (mid-ventricular)	0.662	−0.511 to 0.918	0.072	0.445	−1.662 to 0.867	0.213
FS Segment 17 (apex)	0.564	−0.415 to 0.885	0.093	0.551	−0.581 to 0.885	0.115
LV End diastolic volume	0.919	0.686 to 0.980	0.001			
LV End systolic volume	0.880	0.527 to 0.970	0.002			
Ejection fraction	0.844	0.408 to 0.961	0.005			
Cardiac Output	0.869	0.419 to 0.970	0.006			

ICC: Intraclass Correlation Coefficient.

Table S4. Intraobserver reproducibility of the fetal heart speckle tracking segmental analysis results using 4D-STIC

Variable	ICC	95% confidence interval	ICC	95% confidence interval
		FETAL CARDIAC MORPHOMETRY		
**End-diastolic diameter**				
	Left ventricle		Right ventricle	
Segment 1	0.853	0.696 to 0.929	0.855	0.702 to 0.930
Segment 2	0.888	0.770 to 0.946	0.884	0.760 to 0.944
Segment 3	0.916	0.825 to 0.959	0.906	0.804 to 0.955
Segment 4	0.923	0.839 to 0.963	0.920	0.833 to 0.961
Segment 5	0.920	0.833 to 0.961	0.930	0.854 to 0.966
Segment 6	0.917	0.828 to 0.960	0.938	0.871 to 0.970
Segment 7	0.916	0.827 to 0.960	0.945	0.885 to 0.973
Segment 8	0.917	0.828 to 0.96	0.949	0.894 to 0.975
Segment 9	0.924	0.841 to 0.964	0.936	0.867 to 0.969
Segment 10	0.920	0.835 to 0.961	0.954	0.904 to 0.978
Segment 11	0.922	0.839 to 0.962	0.955	0.907 to 0.979
Segment 12	0.922	0.838 to 0.962	0.955	0.907 to 0.978
Segment 13	0.919	0.832 to 0.961	0.954	0.905 to 0.978
Segment 14	0.915	0.824 to 0.959	0.953	0.903 to 0.977
Segment 15	0.911	0.816 to 0.957	0.952	0.900 to 0.977
Segment 16	0.909	0.812 to 0.956	0.948	0.892 to 0.975
Segment 17	0.912	0.818 to 0.958	0.943	0.881 to 0.972
Segment 18	0.923	0.839 to 0.963	0.938	0.871 to 0.970
Segment 19	0.933	0.861 to 0.968	0.934	0.862 to 0.968
Segment 20	0.938	0.872 to 0.970	0.929	0.852 to 0.966
Segment 21	0.937	0.870 to 0.970	0.924	0.841 to 0.963
Segment 22	0.933	0.862 to 0.968	0.919	0.832 to 0.961
Segment 23	0.930	0.856 to 0.966	0.915	0.824 to 0.959
Segment 24	0.929	0.853 to 0.966	0.912	0.818 to 0.958
**Sphericity index**				
	Left ventricle		Right ventricle	
Segment 1	0.440	−0.201 to 0.736	0.526	−0.042 to 0.872
Segment 2	0.494	−0.015 to 0.752	0.344	−0.368 to 0.685
Segment 3	0.565	0.114 to 0.789	0.457	−0.122 to 0.738
Segment 4	0.582	0.134 to 0.798	0.558	0.093 to 0.786
Segment 5	0.575	0.110 to 0.796	0.624	0.231 to 0.817
Segment 6	0.571	0.099 to 0.795	0.666	0.318 to 0.838
Segment 7	0.591	0.141 to 0.804	0.694	0.375 to 0.851

Segment 8	0.610	0.181 to 0.813	0.701	0.389 to 0.855
Segment 9	0.702	0.392 to 0.855	0.665	0.298 to 0.840
Segment 10	0.664	0.296 to 0.839	0.705	0.396 to 0.857
Segment 11	0.694	0.359 to 0.853	0.702	0.388 to 0.855
Segment 12	0.710	0.392 to 0.861	0.693	0.370 to 0.851
Segment 13	0.729	0.435 to 0.870	0.684	0.350 to 0.847
Segment 14	0.750	0.480 to 0.880	0.676	0.333 to 0.843
Segment 15	0.77	0.522 to 0.889	0.659	0.298 to 0.835
Segment 16	0.779	0.541 to 0.893	0.641	0.24 to 0.827
Segment 17	0.787	0.561 to 0.897	0.609	0.192 to 0.812
Segment 18	0.798	0.584 to 0.902	0.575	0.118 to 0.795
Segment 19	0.903	0.597 to 0.905	0.537	0.036 to 0.777
Segment 20	0.799	0.589 to 0.903	0.499	−0.048 to 0.760
Segment 21	0.793	0.576 to 0.900	0.466	−0.122 to 0.744
Segment 22	0.789	0.566 to 0.897	0.448	−0.163 to 0.736
Segment 23	0.784	0.558 to 0.895	0.437	−0.187 to 0.731
Segment 24	0.782	0.552 to 0.894	0.432	−0.199 to 0.894
		**FETAL CARDIAC FUNCTION**		
**Shortening fraction**				
	Left ventricle		Right ventricle	
Segment 1	0.302	−0.561 to 0.684	0.775	0.526 to 0.894
Segment 2	0.502	−0.049 to 0.764	0.681	0.351 to 0.844
Segment 3	0.569	0.084 to 0.796	0.684	0.358 to 0.846
Segment 4	0.633	0.218 to 0.826	0.667	0.322 to 0.838
Segment 5	0.655	0.279 to 0.834	0.638	0.260 to 0.824
Segment 6	0.700	0.375 to 0.856	0.621	0.221 to 0.816
Segment 7	0.729	0.436 to 0.870	0.635	0.247 to 0.823
Segment 8	0.741	0.460 to 0.876	0.669	0.317 to 0.84
Segment 9	0.748	0.472 to 0.879	0.801	0.579 to 0.906
Segment 10	0.760	0.499 to 0.885	0.817	0.613 to 0.913
Segment 11	0.777	0.535 to 0.892	0.809	0.598 to 0.909
Segment 12	0.790	0.566 to 0.899	0.776	0.530 to 0.893
Segment 13	0.801	0.589 to 0.904	0.729	0.432 to 0.871
Segment 14	0.809	0.607 to 0.908	0.683	0.334 to 0.849
Segment 15	0.814	0.618 to 0.910	0.647	0.254 to 0.833
Segment 16	0.813	0.616 to 0.909	0.624	0.201 to 0.822
Segment 17	0.805	0.599 to 0.906	0.619	0.188 to 0.820
Segment 18	0.794	0.575 to 0.900	0.634	0.221 to 0.827
Segment 19	0.782	0.551 to 0.895	0.654	0.264 to 0.837
Segment 20	0.772	0.530 to 0.890	0.666	0.288 to 0.842

Segment 21	0.765	0.515 to 0.887	0.665	0.288 to 0.842
Segment 22	0.760	0.504 to 0.884	0.612	0.161 to 0.819
Segment 23	0.756	0.496 to 0.882	0.607	0.152 to 0.817
Segment 24	0.754	0.492 to 0.881	0.604	0.146 to 0.815
ICC: Intraclass Correlation Coefficient.				

Table S5. Interobserver reproducibility of the fetal heart speckle tracking segmental analysis results using 4D-STIC.

Variable	ICC	95% confidence interval	ICC	95% confidence interval
		**FETAL CARDIAC MORPHOMETRY**		
**End-diastolic diameter**				
	Left ventricle		Right ventricle	
Segment 1	0.746	0.464 to 0.881	0.891	0.751 to 0.950
Segment 2	0.836	0.659 to 0.921	0.914	0.802 to 0.961
Segment 3	0.856	0.698 to 0.931	0.926	0.828 to 0.966
Segment 4	0.860	0.707 to 0.932	0.933	0.847 to 0.969
Segment 5	0.854	0.698 to 0.929	0.937	0.860 to 0.970
Segment 6	0.847	0.686 to 0.926	0.937	0.865 to 0.970
Segment 7	0.842	0.676 to 0.923	0.933	0.860 to 0.968
Segment 8	0.841	0.671 to 0.923	0.927	0.848 to 0.965
Segment 9	0.841	0.666 to 0.924	0.921	0.835 to 0.962
Segment 10	0.852	0.693 to 0.928	0.920	0.836 to 0.961
Segment 11	0.861	0.713 to 0.933	0.923	0.842 to 0.963
Segment 12	0.869	0.729 to 0.937	0.926	0.846 to 0.964
Segment 13	0.870	0.733 to 0.937	0.925	0.844 to 0.964
Segment 14	0.866	0.723 to 0.935	0.922	0.837 to 0.962
Segment 15	0.858	0.707 to 0.931	0.914	0.822 to 0.959
Segment 16	0.850	0.691 to 0.927	0.902	0.796 to 0.953
Segment 17	0.884	0.675 to 0.925	0.882	0.745 to 0.994
Segment 18	0.841	0.674 to 0.923	0.866	0.725 to 0.935
Segment 19	0.829	0.648 to 0.917	0.845	0.681 to 0.925
Segment 20	0.804	0.598 to 0.905	0.822	0.634 to 0.914
Segment 21	0.776	0.538 to 0.892	0.799	0.588 to 0.902
Segment 22	0.754	0.492 to 0.881	0.781	0.551 to 0.894
Segment 23	0.739	0.461 to 0.874	0.769	0.527 to 0.888
Segment 24	0.730	0.443 to 0.87	0.762	0.513 to 0.884
**Sphericity index**				
	Left ventricle		Right ventricle	

Segment 1	0.390	−0.161 to 0.694	0.333	−0.227 to 0.659
Segment 2	0.548	0.097 to 0.778	0.560	0.103 to 0.786
Segment 3	0.605	0.178 to 0.81	0.624	0.207 to 0.821
Segment 4	0.630	0.217 to 0.823	0.675	0.295 to 0.847
Segment 5	0.625	0.218 to 0.82	0.706	0.355 to 0.862
Segment 6	0.609	0.201 to 0.81	0.717	0.385 to 0.867
Segment 7	0.586	0.169 to 0.797	0.709	0.379 to 0.862
Segment 8	0.575	0.150 to 0.791	0.691	0.353 to 0.851
Segment 9	0.495	−0.110 to 0.754	0.683	0.329 to 0.850
Segment 10	0.604	0.204 to 0.806	0.648	0.286 to 0.829
Segment 11	0.628	0.249 to 0.818	0.635	0.263 to 0.822
Segment 12	0.652	0.295 to 0.830	0.617	0.229 to 0.813
Segment 13	0.666	0.322 to 0.837	0.604	0.201 to 0.806
Segment 14	0.668	0.326 to 0.838	0.602	0.194 to 0.806
Segment 15	0.656	0.303 to 0.832	0.602	0.189 to 0.807
Segment 16	0.630	0.254 to 0.819	0.596	0.169 to 0.805
Segment 17	0.445	−0.101 to 0.728	0.628	0.212 to 0.823
Segment 18	0.495	−0.006 to 0.751	0.552	0.059 to 0.785
Segment 19	0.345	−0.296 to 0.677	0.533	0.016 to 0.777
Segment 20	0.134	−0.719 to 0.573	0.514	−0.024 to 0.768
Segment 21	0.28	−0.507 to 0.657	0.502	−0.044 to 0.761
Segment 22	0.148	−0.800 to 0.596	0.494	−0.056 to 0.757
Segment 23	0.280	−1.548 to 0.374	0.491	−0.060 to 0.755
Segment 24	0.008	−1.118 to 0.531	0.488	−0.063 to 0.753
		**FETAL CARDIAC FUNCTION**		
**Shortening fraction**				
	Left ventricle		Right ventricle	
Segment 1	0.116	−0.951 to 0.595	0.506	−0.055 to 0.772
Segment 2	0.261	−0.595 to 0.646	0.661	0.287 to 0.841
Segment 3	0.299	−0.502 to 0.669	0.666	0.268 to 0.843
Segment 4	0.388	−0.312 to 0.712	0.761	0.421 to 0.893
Segment 5	0.486	−0.100 to 0.757	0.796	0.526 to 0.907
Segment 6	0.648	0.265 to 0.831	0.782	0.541 to 0.895
Segment 7	0.692	0.359 to 0.852	0.740	0.469 to 0.874
Segment 8	0.720	0.420 to 0.865	0.694	0.369 to 0.852
Segment 9	0.742	0.466 to 0.875	0.666	0.303 to 0.839
Segment 10	0.768	0.520 to 0.888	0.669	0.305 to 0.841
Segment 11	0.794	0.573 to 0.901	0.695	0.36 to 0.853
Segment 12	0.813	0.611 to 0.910	0.725	0.429 to 0.868
Segment 13	0.823	0.632 to 0.914	0.746	0.476 to 0.877

Segment 14	0.825	0.637 to 0.915	0.755	0.496 to 0.882
Segment 15	0.820	0.629 to 0.913	0.757	0.501 to 0.882
Segment 16	0.806	0.602 to 0.906	0.754	0.494 to 0.881
Segment 17	0.782	0.544 to 0.894	0.745	0.476 to 0.876
Segment 18	0.748	0.485 to 0.878	0.732	0.453 to 0.870
Segment 19	0.703	0.394 to 0.855	0.605	0.151 to 0.816
Segment 20	0.648	0.288 to 0.828	0.557	0.058 to 0.793
Segment 21	0.594	0.184 to 0.801	0.693	0.331 to 0.859
Segment 22	0.552	0.104 to 0.766	0.670	0.286 to 0.849
Segment 23	0.524	0.050 to 0.766	0.479	−0.082 to 0.754
Segment 24	0.508	0.020 to 0.758	0.649	0.244 to 0.839
ICC: Intraclass Correlation Coefficient.				

Table S6. Comparison of Interobserver reproducibility of the fetal heart speckle tracking segmental analysis results using 2D vs 4D-STIC.

Variable	ICC	95% confidence interval	ICC	95% confidence interval
**FETAL CARDIAC MORPHOMETRY**				
**End-diastolic diameter**				
	Left ventricle		Right ventricle	
Segment 1	0.833	0.529 to 0.930	0.912	0.803 to 0.959
Segment 2	0.849	0.540 to 0.939	0.917	0.793 to 0.963
Segment 3	0.857	0.510 to 0.944	0.919	0.785 to 0.965
Segment 4	0.856	0.498 to 0.945	0.915	0.775 to 0.963
Segment 5	0.855	0.494 to 0.944	0.909	0.768 to 0.960
Segment 6	0.855	0.492 to 0.944	0.905	0.767 to 0.957
Segment 7	0.856	0.485 to 0.945	0.902	0.771 to 0.955
Segment 8	0.858	0.479 to 0.946	0.899	0.775 to 0.953
Segment 9	0.858	0.461 to 0.947	0.825	0.638 to 0.915
Segment 10	0.860	0.425 to 0.949	0.888	0.768 to 0.946
Segment 11	0.863	0.381 to 0.952	0.881	0.755 to 0.942
Segment 12	0.864	0.356 to 0.953	0.873	0.739 to 0.939
Segment 13	0.861	0.370 to 0.951	0.868	0.729 to 0.936
Segment 14	0.858	0.423 to 0.948	0.868	0.729 to 0.936
Segment 15	0.857	0.495 to 0.945	0.871	0.734 to 0.937
Segment 16	0.861	0.578 to 0.944	0.872	0.737 to 0.938
Segment 17	0.871	0.661 to 0.944	0.871	0.753 to 0.938
Segment 18	0.885	0.736 to 0.947	0.866	0.725 to 0.935
Segment 19	0.893	0.776 to 0.948	0.855	0.701 to 0.930

Segment 20	0.889	0.772 to 0.946	0.838	0.662 to 0.922
Segment 21	0.879	0.748 to 0.942	0.818	0.621 to 0.913
Segment 22	0.867	0.723 to 0.936	0.803	0.589 to 0.905
Segment 23	0.859	0.706 to 0.932	0.793	0.568 to 0.901
Segment 24	0.853	0.695 to 0.930	0.787	0.556 to 0.898
**Shpericity index**				
	Left ventricle		Right ventricle	
Segment 1	0.222	−0.401 to 0.601	0.637	0.226 to 0.823
Segment 2	0.222	−0.376 to 0.591	0.689	0.368 to 0.849
Segment 3	0.264	−0.301 to 0.612	0.723	0.432 to 0.866
Segment 4	0.305	−0.240 to 0.636	0.736	0.459 to 0.872
Segment 5	0.340	−0.192 to 0.658	0.748	0.483 to 0.878
Segment 6	0.375	−0.151 to 0.680	0.752	0.493 to 0.880
Segment 7	0.401	−0.124 to 0.696	0.756	0.500 to 0.881
Segment 8	0.416	−0.109 to 0.706	0.752	0.492 to 0.879
Segment 9	0.463	−0.062 to 0.737	0.738	0.457 to 0.873
Segment 10	0.441	−0.090 to 0.723	0.715	0.408 to 0.862
Segment 11	0.458	−0.081 to 0.735	0.689	0.352 to 0.851
Segment 12	0.474	−0.071 to 0.746	0.674	0.317 to 0.843
Segment 13	0.490	−0.049 to 0.755	0.676	0.320 to 0.844
Segment 14	0.520	0.000 to 0.771	0.691	0.352 to 0.852
Segment 15	0.546	0.056 to 0.782	0.706	0.386 to 0.859
Segment 16	0.566	0.115 to 0.789	0.713	0.402 to 0.862
Segment 17	0.589	0.172 to 0.799	0.698	0.370 to 0.855
Segment 18	0.598	0.192 to 0.803	0.666	0.300 to 0.840
Segment 19	0.596	0.175 to 0.804	0.620	0.201 to 0.818
Segment 20	0.577	0.121 to 0.796	0.561	0.075 to 0.790
Segment 21	0.545	0.044 to 0.782	0.508	−0.036 to 0.765
Segment 22	0.519	−0.015 to 0.770	0.477	−0.101 to 0.750
Segment 23	0.501	−0.053 to 0.762	0.459	−0.140 to 0.741
Segment 24	0.491	−0.076 to 0.757	0.452	−0.155 to 0.738
		**FETAL CARDIAC FUNCTION**		
**Shortening fraction**				
	Left ventricle		Right ventricle	
Segment 1	0.643	0.227 to 0.838	0.339	−0.521 to 0.710
Segment 2	0.378	−0.170 to 0.689	0.237	−0.697 to 0.658
Segment 3	0.246	−0.366 to 0.614	0.309	−0.509 to 0.684
Segment 4	0.137	−0.602 to 0.565	0.225	−0.657 to 0.640
Segment 5	0.390	−0.167 to 0.693	0.188	−0.705 to 0.617
Segment 6	0.356	−0.239 to 0.677	0.298	−0.474 to 0.673

Segment 7	0.342	−0.271 to 0.671	0.246	−0.536 to 0.641
Segment 8	0.356	−0.250 to 0.679	0.068	−1.447 to 0.531
Segment 9	0.387	−0.196 to 0.695	0.160	−0.769 to 0.607
Segment 10	0.410	−0.160 to 0.708	0.171	−0.794 to 0.616
Segment 11	0.407	−0.178 to 0.708	0.142	−0.855 to 0.597
Segment 12	0.384	−0.246 to 0.699	0.269	−0.580 to 0.656
Segment 13	0.367	−0.309 to 0.694	0.371	−0.347 to 0.703
Segment 14	0.382	−0.301 to 0.704	0.436	−0.190 to 0.732
Segment 15	0.422	−0.224 to 0.724	0.470	−0.108 to 0.747
Segment 16	0.466	−0.117 to 0.744	0.480	−0.085 to 0.752
Segment 17	0.501	−0.021 to 0.758	0.480	−0.091 to 0.752
Segment 18	0.519	0.035 to 0.764	0.479	−0.105 to 0.753
Segment 19	0.629	0.242 to 0.821	0.464	−0.146 to 0.747
Segment 20	0.524	0.233 to 0.818	0.429	−0.226 to 0.731
Segment 21	0.615	0.217 to 0.814	0.385	−0.320 to 0.710
Segment 22	0.608	0.204 to 0.811	0.351	−0.391 to 0.694
Segment 23	0.596	0.159 to 0.809	0.330	−0.437 to 0.684
Segment 24	0.595	0.156 to 0.808	0.327	−0.462 to 0.678

ICC: Intraclass Correlation Coefficient.

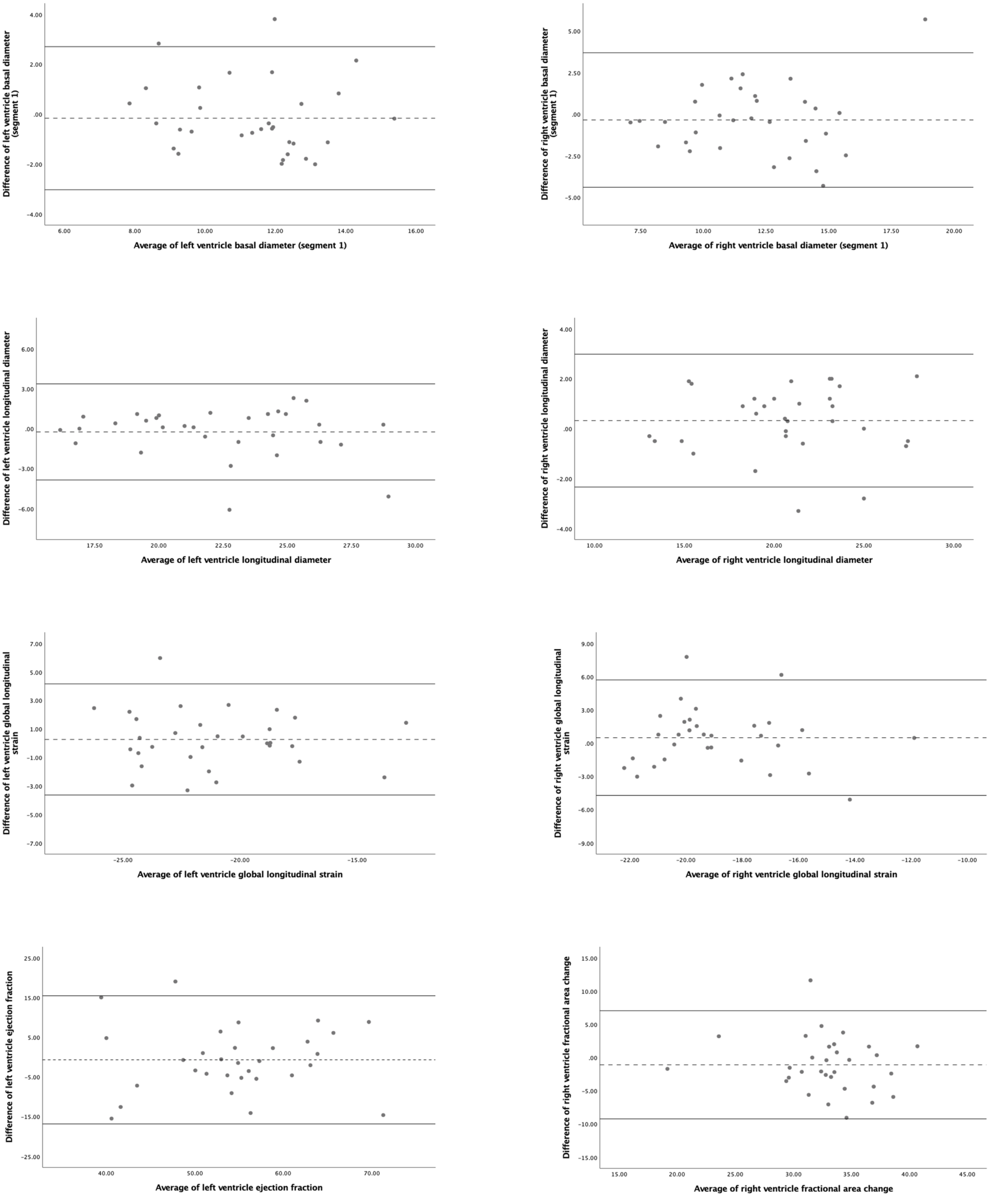

Figure S1. Bland-Altman plots for intraobserver reproducibility of fetal heart speckle tracking analysis of morphometric and functional parameters using 4D-Spatio Temporal Image Correlation (4D-STIC). Upper figures: left ventricle basal diameter (segment 1) (left) and right ventricle basal diameter (segment 1) (right); Middle upper figures: left ventricle longitudinal diameter (left) and right ventricle longitudinal diameter (right); Middle bottom: left ventricle global longitudinal strain (left) and right ventricle global longitudinal strain (right); bottom figures: left ventricle ejection fraction (left) and right ventricle fractional area change (right).
